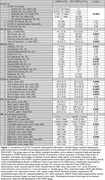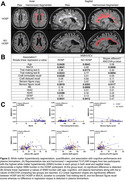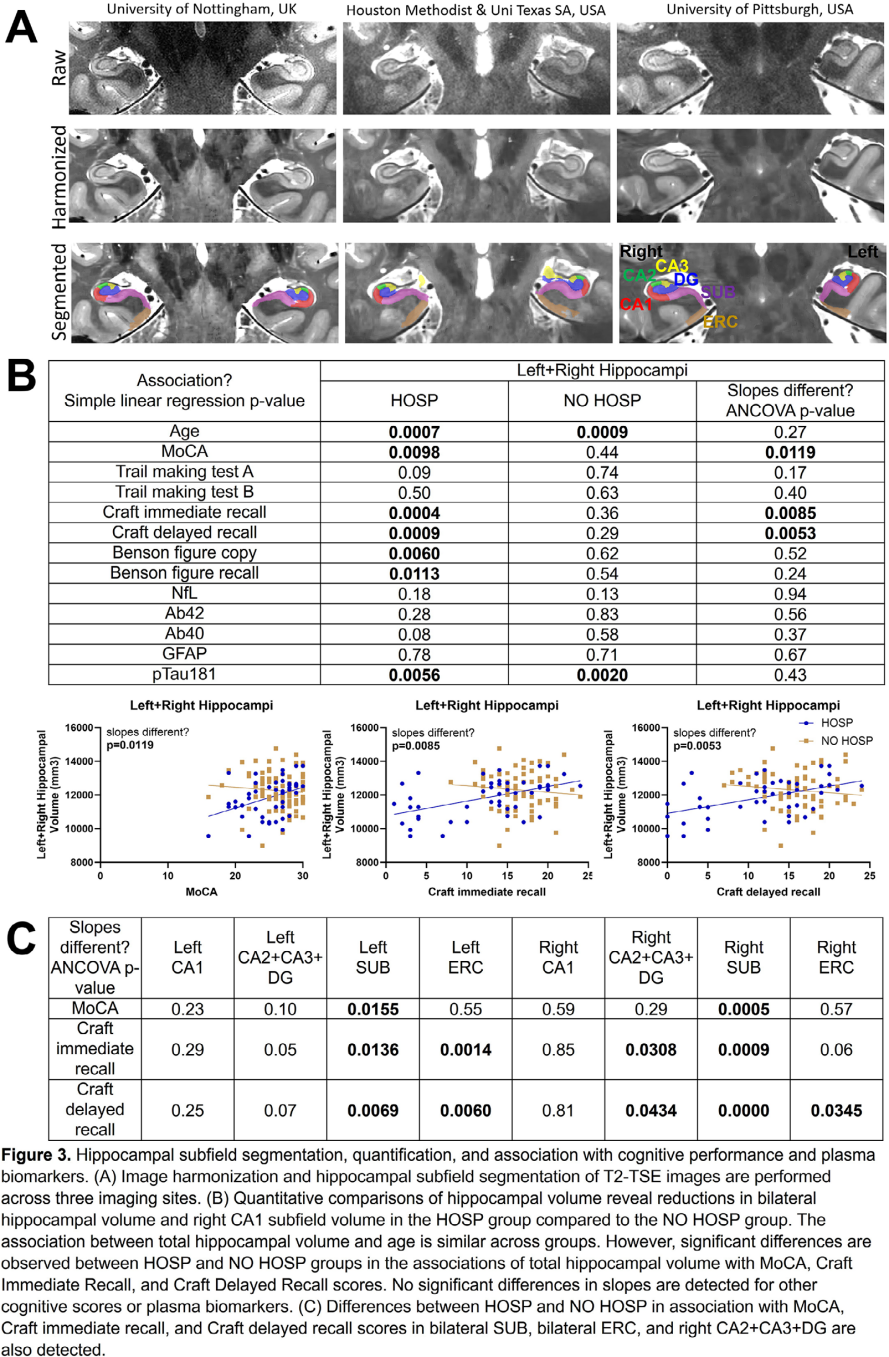# Examining White Matter Hyperintensities, Hippocampal Subfields, Cognitive Function, and Plasma Biomarkers in Recovered COVID‐19 Patients

**DOI:** 10.1002/alz70856_098083

**Published:** 2025-12-24

**Authors:** Jr‐Jiun Liou, Tales Santini, Jinghang Li, Monica Goss, Tiffany F. Kautz, Julie Parker‐Garza, Vibhuti N Patel, Oluwatobi F Adeyemi, Gabriel A. de Erausquin, Valentina R. Garbarino, Mohamad Habes, Jayandra Jung Himali, Christof Karmonik, Beth E. Snitz, Joseph M Mettenburg, Minjie Wu, Howard J Aizenstein, Anna Marsland, Peter Gianaros, Richard Bowtell, Olivier Mougin, Penny Gowland, Mohammad Zia U.H Katshu, Farhaan S Vahidy, Timothy D. Girard, Heidi I.L. Jacobs, Akram A. Hosseini, Sudha Seshadri, Tamer S Ibrahim

**Affiliations:** ^1^ University of Pittsburgh, Pittsburgh, PA, USA; ^2^ University of Texas Health San Antonio, San Antonio, TX, USA; ^3^ Sir Peter Mansfield Imaging Centre, University of Nottingham, Nottingham, Nottinghamshire, United Kingdom; ^4^ Houston Methodist Research Institute, Houston, TX, USA; ^5^ University of Nottingham, Nottingham, United Kingdom; ^6^ School of Medicine, University of Nottingham, Nottingham, Nottinghamshire, United Kingdom; ^7^ Massachusetts General Hospital, Boston, MA, USA

## Abstract

**Background:**

Evidence suggests COVID‐19 impacts brain health, with clinical MRI revealing neurologic manifestations but no consistent pattern. This study examines differences in research MRI neuroimaging biomarkers, cognitive function, and plasma biomarkers between previously hospitalized and non‐hospitalized recovered COVID‐19 patients.

**Methods:**

Between May 2020 and March 2024, 179 participants with no dementia history from three U.S. and one U.K. medical centers completed 7T MRI neuroimaging, cognitive assessments, and blood collection. Cognitive domains, including mild cognitive impairment (MoCA), verbal memory (Craft immediate/delayed recall), visual memory (Benson figure recall), psychomotor speed (Trail making test A/B), and visuospatial construction (Benson figure copy), were measured using NACC Neuropsych Battery UDS3 IVP C2. Plasma biomarkers (NfL, Ab42, Ab40, GFAP, pTau181) were quantified. Harmonized T1‐MP2RAGE, T2‐FLAIR, and T2‐TSE images enabled segmentation for whole‐brain morphometry, white matter hyperintensity (WMHs), and hippocampal subfields. After image quality control, 163 participants were analyzed. Unpaired t‐tests compared groups (hospitalized [HOSP, *n* = 52, 19.0±7.5 months post‐discharge] vs. non‐hospitalized [NO HOSP, *n* = 111]), and linear regressions examined associations between imaging biomarkers, cognitive scores, and plasma biomarkers, with ANCOVA assessing regression slope differences.

**Results:**

HOSP patients showed similar global WMH burden but smaller hippocampal volumes (total, right CA1) and lower cognitive performance (MoCA, Trail Making Test B, Craft immediate/delayed recall, Benson figure copy) and lower plasma GFAP compared to age‐matched NO HOSP (Figure 1). Associations of WMH burden with MoCA (*p* = 0.0006), duration completing Trail making test B (*p* = 0.0054), and Benson figure recall (*p* = 0.0117) differed significantly between HOSP and NO HOSP while no plasma biomarker differences were detected (Figure 2). In the hippocampus, the associations of total and subfield volumes with MoCA (*p* = 0.0119) and Craft immediate (*p* = 0.0085) and delayed (*p* = 0.0053) recall also differed significantly between groups whereas no difference in plasma biomarkers was detected (Figure 3).

**Conclusions:**

In this multinational 7T MRI cohort, hospitalization for COVID‐19 was associated with worse cognitive performance, smaller hippocampal volumes, and distinct relationships between imaging and cognitive markers but not plasma biomarkers, highlighting lasting neurological impacts of severe illness. Longitudinal assessments and the inclusion of other illnesses, such as pneumonia and sepsis, could clarify the impact of COVID‐19 hospitalization on overall brain health.